# Probing Executive Function in the Toddler Data Desert: A Touchscreen fNIRS Study of Working Memory and ADHD-Related Symptoms

**DOI:** 10.3390/bs16101894

**Published:** 2026-10-14

**Authors:** Anastasia N. Kerr-German, Eileen Lin, Grace Gasaway, Emily Lundstrum, Samuel Patton, Haylee Hudson, Caitie Busch

**Affiliations:** 1 Department of Psychology, Mercer University, Macon, GA 31207, USA; 11036071@live.mercer.edu (E.L.); grace.nealie.gasaway@live.mercer.edu (G.G.); samuel.harris.patton@live.mercer.edu (S.P.); 2 Department of Psychology, University of Tennessee, Knoxville, TN 37996, USA; 3 Nebraska Children’s Hospital, Omaha, NE 68114, USA; hhudso12@vols.utk.edu; 4 Psychiatric Residential Treatment Facility, Boys Town National Research Hospital, Boys Town, NE 68010, USA; caitlynbusch@unomaha.edu

**Keywords:** fNIRS, working memory, dimensional attention, ADHD risk, early childhood

## Abstract

Attention-deficit/hyperactivity disorder (ADHD) is associated with early differences in attentional control and working memory (WM), yet relatively few paradigms are developmentally feasible across the toddler-to-preschool period while also distinguishing spatial from featural information use. Addressing this “Toddler Data Desert,” we extended a self-ordered pointing paradigm into a touchscreen WM search task paired with functional near-infrared spectroscopy (fNIRS) to characterize developmental changes in search behavior and cortical recruitment. The larger study enrolled 204 children aged 2–6 years; 200 completed both Set Size 3 conditions, 197 completed both Set Size 6 conditions, and 162 completed both Set Size 9 conditions after a performance-based advancement criterion. Children completed stationary, spatially informative and feature-informative, featural/color-informative Treasure Chest blocks across increasing memory loads while fNIRS measured bilateral frontal, temporal, and parietal responses. Behaviorally, children required fewer selections during spatial than featural search at Set Sizes 3 and 6, and performance improved with age; this condition difference was no longer statistically reliable at Set Size 9 within the selected higher-performing subset. Immediate perseverative selections also decreased markedly with age. Exploratory post hoc sequence analyses further showed increasingly organized spatial search under higher memory load, whereas featural search remained comparatively heterogeneous, and organization in a preceding spatial block did not reliably predict subsequent featural search. Symptom-defined ADHD risk did not significantly predict behavioral efficiency. Neurally, spatial and featural blocks showed age-associated differences in distributed frontotemporal–parietal recruitment, and the elevated-symptom group showed broader frontal recruitment despite comparable behavioral performance. Together, these findings support the Treasure Chest Task as a toddler-feasible approach for examining developmental differences in spatial and featural working-memory search while highlighting the value of integrating behavioral sequence measures with cortical activity. Neural risk-group findings should be interpreted as cross-sectional associations rather than diagnostic biomarkers.

## 1. Introduction

Attention-deficit/hyperactivity disorder (ADHD) is characterized by persistent patterns of inattention and/or hyperactivity–impulsivity that interfere with functioning (American Psychiatric Association, 2022). ADHD is among the most prevalent neurodevelopmental disorders in childhood. A growing literature suggests that working-memory and attentional-control differences are important components of the ADHD phenotype and may be detectable before school age (Castellanos & Tannock, 2002; Kofler et al., 2018; Martinussen et al., 2005). In the present study, however, “ADHD risk” refers to elevated-symptom risk based on parent-report screening and familial information; it does not indicate a clinical diagnosis in the child.

Visual attention allows the viewer to selectively attend to a subset of the visual world and prioritize information for further processing. Thus, visual attention operates within a capacity-limited working memory system to maintain an alert state, orient to external events, and self-regulate responses. Alerting, orienting, and self-regulation form the foundation for higher-level executive attention, which emerges in late infancy and early childhood. When delayed, such development can signal a risk of psychopathology, including ADHD (Kofler et al., 2011; Kasper et al., 2012; Luo et al., 2019; Johnson et al., 2008; Diamond, 2013; Hanania & Smith, 2010). A key aspect of executive attention involves not only attending to objects but attending to specific features of objects in a goal-directed way. For example, attention to an object’s spatial location appears to develop earlier than attention to its intrinsic features, such as color (Calub et al., 2022; Diamond, 2013). This specific component of attentional development has not been explored in very young children with elevated ADHD-related symptoms or familial risk. Attentional selectivity to objects has been correlated with other executive functions, including inhibition and working memory, in early life (Diamond, 2013). However, more nuanced associations unpacking how selectivity interacts with specific components of working memory, such as capacity or proactive strategy use, over the course of development are still not fully understood, limiting our ability to characterize early risk-associated cognitive patterns without overextending them as diagnostic indicators.

### 1.1. Separate Developmental Trajectories for Attention to Extrinsic and Intrinsic Features

The development of selectivity to spatial and featural information in the visual world is gradual over the first 5 years of life and continues into middle childhood (Hanania & Smith, 2010; Conejero & Rueda, 2017; de Jong et al., 2015; Gaertner et al., 2008). Many common tasks used to assess working memory capacity in infancy and early childhood require either color–item or color–spatial bindings (Ross-Sheehy et al., 2003; Simmering, 2012; Cowan et al., 2010, 2011). Therefore, measured improvements in working-memory performance during this period may be due either to improvements in the ability to selectively attend to relevant information (e.g., spatial location or color), domain general increases in working memory capacity, or both. Distinguishing between selective attention and capacity will lead to a better understanding of the underlying sources of working memory deficits in atypical populations, such as children with attention-deficit/hyperactivity disorder (ADHD).

### 1.2. Working Memory Capacity Estimates in Early Development

Older children’s ability to control visual attention depends on whether the task is within their working memory capacity (Cowan et al., 2010). The size and complexity of the visual scene must be considered when assessing toddlers’ attentional control. Both capacity (i.e., the number of items which can be attended) as well as the ability to control attentional resources increase across development (Ross-Sheehy et al., 2003; Simmering, 2012; Cowan et al., 2011). However, attempts to quantify these increases during development have revealed discontinuities in working memory capacity estimates.

The first discontinuity arises between infancy and toddlerhood where differences in task design hinder direct comparisons of results across development. Infant working memory measures often rely on spontaneous oculomotor behaviors such as looking time and gaze direction, suggesting that capacity increases even during the first year of life, but it cannot be compared directly to tasks used with older children and adults (Ross-Sheehy et al., 2003; Simmering, 2012; Cowan et al., 2011). The second discontinuity appears toward the end of toddlerhood because many toddlers and young children struggle to understand the instructions for the standard simultaneous visual array of tasks often used to estimate capacity in older children and adults. For example, nearly half of typically developing (TD) 3-year-olds and one third of TD 5-year-olds failed to complete child-friendly paradigms (Simmering, 2012). If only the highest-performing children can follow instructions, then capacity estimates for this age range will be artificially inflated. Even when infants and young children can complete variations of a visual array task, the number of items in the array influences infants’ and children’s capacity estimates more than adults’ capacity estimates (Ross-Sheehy et al., 2021; Ross-Sheehy & Eschman, 2019). These findings highlight the need to separate attentional selectivity from capacity when assessing these hard-to-test age groups. This measurement challenge extends into older children with ADHD.

Aside from capacity growth, individual strategy use also contributes to developmental and individual variability in working-memory performance. A working memory strategy is any technique that facilitates storage and/or processing (Turley-Ames, 2003). Even by the age of five, children’s use of simple strategies predicts recall (AuBuchon et al., 2022), and the ability to implement appropriate strategies continues to shape working-memory performance into adulthood (Fellman et al., 2020; Forsberg et al., 2020). Most investigations of strategy use—in both adults and children—focus on strategies typically used to bind items to temporal locations (i.e., the order of item presentation) rather than strategies used to maintain purely spatial features or purely item-feature information (i.e., color) independent of spatial or temporal bindings. However, initial work suggests that adults can use both adjacent spatial locations and color similarities to strategically organize visual arrays with the greatest benefits coming when the strategy involves spatial adjacencies (Peterson et al., 2015; Woodman et al., 2003).

To describe both typical and atypical attentional control from the toddler years through early childhood, we need a task that fits the following criteria: First, the task must be feasible for children aged 2–6. Second, the task must show control for developmental changes in capacity. Third, the task must independently assess spatial and featural attention with consistent instructions and response formats. The existing measurement discontinuities highlight the importance of integrating neural measures that can help clarify attentional control and working memory processes when behavioral performance is difficult to interpret.

### 1.3. Neural Underpinnings of Selective Attention and Rule Use in Young Children

Attentional control refers to the selective allocation of cognitive resources to external visual stimuli (Diamond, 2013). This process involves filtering competing inputs and resolving the balance between bottom-up and top-down processes (e.g., frontal and parietal areas with associated subcortical regions), creating attention (Hanania & Smith, 2010). Previous studies have shown there are implications with bottom-up processes in individuals with ADHD. Studies found that individuals with ADHD are significantly slower and produce more varied responses that are less accurate than the TD peers (Klein et al., 2025).

The parietal cortex plays an important role in orienting and alerting through its involvement in both dorsal (frontal eye fields, superior parietal lobe) and ventral (temporoparietal junction, ventral frontal cortex) attention streams. These systems support visuospatial orienting and reorienting and overlap with broader frontoparietal networks that support executive control (Ross-Sheehy et al., 2003, 2021; Simmering, 2012; Cowan et al., 2010, 2011; Ross-Sheehy & Eschman, 2019). Recent toddler and preschool fNIRS work reinforces the feasibility of studying these networks early in development: longitudinal data show right inferior frontal and parietal recruitment during emerging inhibitory control (Fiske et al., 2025), and prefrontal intrinsic functional connectivity is associated with executive-function performance in 4- to 5-year-old children (Eng et al., 2025). Across development, increasing coordination and specialization of these systems may support more efficient attentional control and working-memory performance.

### 1.4. Selective Attention and Working Memory in ADHD

Across their lifespans, individuals diagnosed with ADHD show group-level differences in attention and working memory (Kofler et al., 2011; Kasper et al., 2012; Luo et al., 2019; Johnson et al., 2008). In older children, recent fNIRS studies likewise report altered prefrontal recruitment during executive tasks, although the direction and localization of effects vary with task demands and analytic features (Bian et al., 2025; Chu et al., 2025). These findings underscore that cortical recruitment differences are not diagnostic in isolation and should not be extrapolated directly from school-age clinical samples to symptom-defined risk in toddlers. Further, little is known about when such differences first emerge or how early attentional control and working memory jointly contribute to later impairment. Thus, more work characterizing potential patterns in developmentally scalable tasks in much younger children are needed to map the cross-sectional ontogeny of this disorder prior to the age of diagnosis (Kieling et al., 2010). Building a robust literature for toddlers will better guide future longitudinal works.

Recent studies have begun to clarify the relationship between working memory and inhibitory control in children with ADHD. Findings showed that increasing working-memory demands disrupts inhibitory control performance, whereas increasing inhibition demands does not affect working memory (Kofler et al., 2024). These results suggest that working memory deficits may represent an underlying mechanism in executive dysfunction observed in individuals with ADHD.

Individual differences in attentional processing are linked to working memory outcomes in young children (Gade et al., 2017; Rapport et al., 2001). Children with stronger attentional control and efficiency tend to perform better working memory tasks, and this association holds strong even among children with ADHD (Kasper et al., 2012). This suggests that attention may moderate working-memory performance regardless of clinical status.

ADHD carries considerable public health significance. Roughly half of the children diagnosed with ADHD in longitudinal clinical cohorts continue to meet diagnostic criteria into adulthood, while an additional subset may show persistent functional difficulties despite no longer meeting the full criteria (Molina et al., 2009; Sibley et al., 2012, 2016, 2017). Yet, early-childhood behavioral criteria do not directly index working-memory or attentional-control mechanisms, and relatively little work has examined neurocognitive correlates before school age.

Working memory is one of the primary executive functions affected in children with ADHD (Friedman et al., 2022). In Westerberg et al. (2004), a main sample of 80 boys aged 8.0–15.3 years (27 with clinically diagnosed ADHD and 53 controls; both groups *M* age = 11.4 years) showed poorer visuospatial working-memory performance in the ADHD group, with a larger group difference among older children. This single study should be interpreted within the broader literature rather than as evidence of a universal developmental trajectory. Working memory serves as a limited-capacity mental workspace in which information is temporarily maintained and manipulated for ongoing cognition. Theoretical perspectives differ in the degree to which ADHD-related impairment is attributed to attention, working memory, or broader executive control (Kerr-German et al., 2022b; Baddeley, 1998; Zelazo et al., 2003). Because working memory is capacity-limited, successful encoding and maintenance depend on attentional and inhibitory processes (Baddeley, 1998; Cowan, 1999; Engle et al., 1999), and developmental delays in these interacting processes may contribute to ADHD-related dysregulation (Konrad et al., 2006).

The current state of the field on working memory dysfunction in ADHD creates challenges when studying children at risk for ADHD prior to school age. Yet, recent theoretical advances in understanding object processing within the framework of executive functioning provide new insights into potential mechanisms by which early attentional processes in at-risk populations may influence later working memory development (Konrad et al., 2006; Buss & Kerr-German, 2019). While theories of executive function are beginning to articulate the relationship among attention, inhibitory control, and working memory in TD children under 5 years old (Buss & Kerr-German, 2019), developmental psychopathology has not systematically or theoretically applied this work to at-risk populations for ADHD.

### 1.5. Current Approach

The current study had three aims: (1) to assess working-memory search in toddlers and preschool-aged children, (2) to use a single paradigm that remains sensitive to developmental changes in attentional selectivity and capacity demands, and (3) to examine whether symptom-defined and familial indicators of ADHD risk are associated with behavioral performance or cortical recruitment in children aged 2–6 years. We administered the Treasure Chest Task to community- and clinic-recruited children while recording hemodynamic responses over bilateral prefrontal, temporal, and parietal cortices using functional near-infrared spectroscopy (fNIRS) (Chen et al., 2020).

The Treasure Chest Task extends earlier stationary/shuffled self-ordered pointing paradigms (Diamond et al., 1997; Pike et al., 2013) rather than replacing them. Its specific contribution is to preserve the same touchscreen response format, goal, and basic instructions while changing whether the spatial location or item color is informative, allowing spatial and featural search to be compared within a toddler-feasible task. The same paradigm also manipulates the set size (3, 6, and, for an eligible subset, 9) to examine performance as memory demands approach or exceed capacity, and pairs these behavioral measures with fNIRS across a broader frontotemporal–parietal montage. Thus, the novelty lies in the combination of developmentally consistent response demands, within-task spatial-versus-featural manipulation, graded capacity demand, and simultaneous cortical measurement in 2- to 6-year-olds. Because shuffled featural trials necessarily include spatial rearrangement and a slightly longer minimum interselection interval than stationary trials, condition differences are interpreted as contrasts in the availability of spatial versus featural identity information rather than as a perfectly isolated single-feature manipulation.

### 1.6. Hypotheses

Prior work suggests that attention to spatial information develops earlier than featural attention (Kofler et al., 2011; Kasper et al., 2012; Luo et al., 2019; Johnson et al., 2008). In other words, spatial attention will be more developed than featural attention in younger children. Thus, younger children will show a stronger recruitment of the parietal cortex during spatial attention. In contrast, older groups will recruit more of the frontal cortex.

We predicted that older children would outperform younger children across both featural and spatial trial blocks and that spatially informative trials would be completed more efficiently than featural/color trials. We further expected age-related differences in cortical recruitment, with greater frontal involvement among older children. Because the risk classification was symptom-based rather than diagnostic, analyses of ADHD risk were treated as tests of group association: we predicted that elevated-symptom risk might be associated with behavioral and/or neural differences relative to the lower-risk group, without treating any observed neural pattern as an ADHD biomarker.

We further hypothesized that visual attentional control would influence children’s search behavior (Kofler et al., 2011; Kasper et al., 2012; Luo et al., 2019; Johnson et al., 2008). The present analysis operationalized one specific prespecified search-control measure: immediate perseveration, defined as repeating the same location or color on the next selection. Although adjacent spatial clustering and color-grouping patterns are theoretically relevant (Woodman et al., 2003), these broader sequence patterns were not part of the prespecified primary analysis. They were subsequently examined in an explicitly exploratory post hoc sequence analysis and are interpreted separately as hypothesis-generating. Accordingly, immediate perseveration remains the primary prespecified search-control outcome, whereas the additional sequence metrics are treated as exploratory.

We hypothesized that during spatial blocks, younger children would primarily rely on the parietal cortex, whereas the older children would engage in more of the frontal cortex. This developmental shift reflects a transition from bottom-up stimulus-driven processing in younger children to top-down rule-directed control in older children. In contrast, during featural blocks, we expected all children to recruit feature-space mapping areas in the temporal and parietal cortices, with younger children relying more heavily on these areas. Older children were predicted to engage frontal regions, reflecting the complete emergence of a dimensional attention network (Buss & Kerr-German, 2019; Kerr-German et al., 2022a). Lastly, we expected that interactions between age and feature type would determine the attentional strategies children used which would in turn influence the pattern of cortical activation during the task.

## 2. Material and Methods

### 2.1. Participants

The current task was part of a larger fNIRS study examining executive-function development in children with varying levels of ADHD-related risk. A total of 204 children were enrolled in the larger study. Eligibility criteria included age 2–6 years, normal or corrected-to-normal vision without known color blindness, typical hearing by parent report, and no known cognitive or neurodevelopmental conditions expected to preclude task participation (e.g., epilepsy, Down syndrome, intellectual disability, or autism spectrum disorder). The present risk grouping reflects elevated parent-reported hyperactivity symptoms and does not constitute an ADHD diagnosis.

Children were recruited through two primary methods. First, eligible families already listed in a research-participant database maintained by the hospital were invited to participate; inclusion in this research database was not itself used as evidence of ADHD diagnosis or risk. Second, to enrich the sample for familial ADHD risk, additional families were identified through hospital medical records when a biological parent or sibling had a documented ADHD diagnosis. Parental informed consent and continuous verbal assent from the child were obtained before participation. Familial diagnostic history was used for recruitment enrichment when available, whereas the child’s primary analytic risk classification was based on the SDQ Hyperactivity subscale described below.

Of the 204 children enrolled in the larger study, 200 completed both Set Size 3 conditions (age 2: *n* = 38; age 3: *n* = 48; age 4: *n* = 57; age 5: *n* = 57). Three additional children did not complete both Set Size 6 conditions, leaving 197 children with complete Set Size 6 data (age 2: *n* = 37; age 3: *n* = 47; age 4: *n* = 57; age 5: *n* = 56). Among these children, 163 met the advancement criteria and began Set Size 9, and 162 completed both Set Size 9 conditions. The reduction between Set Sizes 6 and 9 primarily reflects the protocol-based advancement rule rather than conventional study attrition. Participant retention by age and task stage is summarized in Supplementary Table S1. Available child-care/preschool setting information is summarized in Supplementary Table S2.

Exploratory neural analyses are also included for 86 children who met the study’s elevated-symptom risk criterion of SDQ Hyperactivity ≥ 7 (Brown & Harvey, 2018). Parents also completed the Adult ADHD Self-Report Scale (ASRS-v1.2) to characterize parental symptoms and hereditary risk. The preschool SDQ has established evidence in preschool-aged samples, and newer psychometric work in 1- to 2-year-olds supports its utility particularly for externalizing symptoms while emphasizing that clinically interpretable cutoffs in the youngest ages require further validation (Croft et al., 2015; Byrne et al., 2024). Accordingly, analyses involving the youngest children should be interpreted as dimensional symptom-risk analyses.

### 2.2. Procedures

All procedures were approved by the Boys Town National Research Hospital Institutional Review Board for Research with Human Subjects. Questionnaires were completed by biological parents on a 7th Generation Apple iPad using REDCap over the laboratory’s secure network. During data collection, children sat in a highchair or comfortable child-fitted chair facing a 23.5-inch touchscreen monitor. Room lights were dimmed to support screen visibility and engagement. Children wore a size-appropriate fNIRS EasyCaps (50, 52, or 54 cm) for the NIRSport2 system based on head circumference. Polhemus digitization was completed for 31 children (*n* = 31) across the 4 age groups to test the reliability of the cap placement generated through NIRStar for all participants. Two-year-olds were less likely than older children to tolerate the digitization procedure; therefore, participant-specific image reconstruction was not used in the reported analyses. In all participants, nasion, inion, and vertex landmarks were used to align the cap consistently. Cortical labels reported for channels therefore reflect the standardized probe placement/sensitivity profile based on EasyCap Placements and NIRStar digitization files.

#### Treasure Chest Task

The Treasure Chest Task is a computerized self-ordered pointing paradigm adapted from the stationary and shuffled featural 3-box and 6-box tasks previously used with preschoolers to assess visual working memory (Diamond et al., 1997; Pike et al., 2013). In each trial, children are shown drawings of 3, 6, or 9 open treasure chests, each containing treasure. After, the chests close, and the child is instructed to “click each chest to find the treasure inside,” with the goal of locating all treasures using as few selections as possible.

During stationary blocks, all treasure chests appear identical, so once the chests are closed, spatial location is the primary cue to chest identity. In shuffled featural blocks, each chest is assigned a distinct color (Set Size 3: yellow, red, and green; Set Size 6: yellow, red, green, pink, purple, and light blue; Set Size 9: yellow, red, green, pink, purple, light blue, light green, dark blue, and orange), and the chests change positions after each selection. Children were not required to name the colors; color functioned as a visual identity cue. Thus, lexical knowledge of color names was not required, although successful performance did depend on perceptual color discrimination. Known color blindness was an exclusion criterion.

In addition to the original set sizes of 3 and 6, the current version included the set size of 9 to examine strategy use and neural patterns under super-capacity demands. Only children who successfully completed both stationary and shuffled featural versions of Set Size 6 were eligible to attempt Set Size 9. Following the precedent, children were given a maximum of 20 selections to complete each task version (Pike et al., 2013). However, to reduce frustration levels for a larger battery of tasks administered (i.e., quitting mid-block when failing and refusing to continue the study), children who required more than 18 selections on the shuffled featural Set Size 6 were not advanced to Set Size 9. Accordingly, 200 children completed both Set Size 3 conditions, 197 completed both Set Size 6 conditions, 163 met the advancement criterion and began Set Size 9, and 162 completed both Set Size 9 conditions. Set Size 9 stationary and shuffled featural blocks were administered in that order.

**Figure 1:**
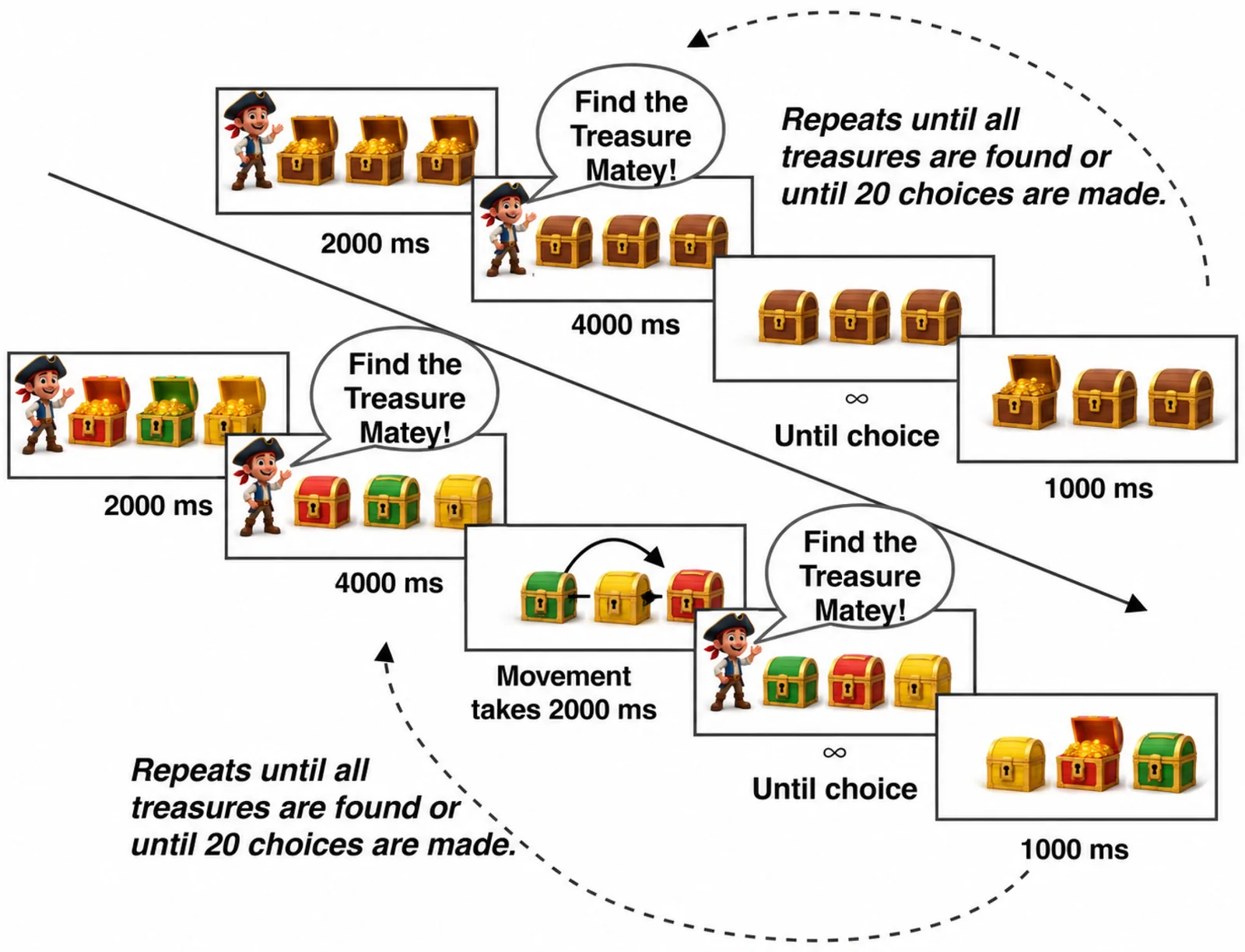
Schematic of the stationary and shuffled featural versions of the treasure chest task with Set Size 3.

On stationary trials, children saw 3, 6, or 9 treasure chests in one, two, or three rows respectively around the center of the monitor (see Figure 1). A pirate image appeared in the upper left corner of the monitor and an audio over the speakers instructed, “Find the treasure, matey.” The child then selected a chest by touching it on the screen. Each chest was left open for 1 s to reveal its contents. If the chest had not been selected previously in that trial, treasure appeared; however, if it had already been previously opened, the chest opened with no treasure. After closing the chest again, the pirate reappeared, and the instructions were repeated, and the child made the next selection. The trials continued until the child found all the treasures (3, 6, or 9 depending on set size) or reached 20 selections (Pike et al., 2013).

Shuffled trials followed the same structure as the stationary trials, except after each instruction to “find the treasure, matey,” the chests rearranged positions on the screen. Specifically, either the first column shifted to the end with the remaining columns moving one position to the left, or the third column shifted to the beginning with all other chests moving one position to the right, The direction of movement was randomized for each selection. This movement lasted for 2 s. As a result, the minimum delay between selections was 5 s for stationary trials and 7 s for shuffled featural trials.

### 2.3. Survey Analyses

Associations among children’s SDQ Hyperactivity scores, age, and parental ASRS Part A scores were assessed using Pearson correlations. Group differences in these measures were examined with t tests. Parental ASRS scores were used as a convergent familial-risk indicator rather than as a diagnostic classification. When information from both biological parents was available, parent symptom scores were examined as specified in the supplemental analyses.

#### 2.3.1. SDQ

Symptom-defined ADHD risk classification was based on the SDQ Hyperactivity subscale. Parents or legal guardians responded to 25 statements as “Not True,” “Somewhat True,” or “Certainly True” (coded 0, 1, and 2; Anbarasan et al., 2020), yielding five subscales and a total difficulty score. The five-item Hyperactivity subscale was used as an index of elevated ADHD-related symptoms. Scores ≥ 7 were classified as elevated-symptom risk and scores 0–6 as lower risk (Brown & Harvey, 2018). This grouping is a screening classification only and should not be interpreted as a diagnosis. Although the SDQ is well established in preschool-aged children (Croft et al., 2015), recent evidence in 1- to 2-year-olds indicates acceptable psychometric properties—particularly for externalizing symptoms—while also noting that clinical cutoffs in very young children remain less well established (Byrne et al., 2024). This limitation is considered in the interpretation of results from the youngest participants.

#### 2.3.2. ASRS 1.2

Biological parents completed the ASRS 1.2 to characterize parental ADHD-related symptoms and hereditary risk, rather than to establish a parent diagnosis within this study. The ASRS includes 18 questions divided into Part A (six questions) and Part B (12 questions), rated from 1 (Never) to 5 (Very Often). Endorsement of four or more Part A symptoms in the clinical scoring range is commonly used as a positive adult ADHD screen (Anbarasan et al., 2020), but a screening score is not equivalent to a clinical diagnosis. When possible, both biological parents were invited to complete the ASRS independently. In the first cohort, nine second-parent surveys were not returned, all from fathers; in the second cohort, 64 were not returned—63 from fathers and one from a mother. Documented ADHD diagnoses in a biological parent or sibling were available for the familial-risk recruitment pathway, but diagnostic family-history information was not uniformly available for every participant and therefore did not replace the child’s SDQ-based analytic grouping.

### 2.4. Behavioral Analyses

Behavioral efficiency was indexed by the number of selections required to complete each block/condition. Non-selection touches (touches to on-screen white space) accounted for less than 1% of all selections and were excluded; their frequency did not differ significantly by risk group or age bin. Each valid selection was additionally coded as new or immediately perseverative. A perseverative error was defined as selecting the same location (spatial block) or the same color identity (featural block) on the immediately subsequent choice. This measure captures immediate repetition but does not quantify other possible search strategies such as adjacency-based spatial clustering, systematic row/column scanning, or higher-order color grouping.

Following the primary behavioral analyses, we conducted exploratory post hoc sequence analyses using concatenated choice-level records from both study cohorts. Choice labels were harmonized across cohorts using the study coding schema. For spatial blocks, item identities were represented by their fixed spatial positions; for featural blocks, color identities were mapped onto their corresponding coding-schema indices, permitting evaluation of spatial organization despite physical rearrangement of the chests. *First-visit order* was defined as the ordered sequence in which each unique location or color identity was selected for the first time, with subsequent repetitions omitted from this sequence. At Set Size 3, an exact *linear edge-to-edge sweep* consisted of left–center–right or right–center–left first visits; *center-first* sequences began at the center and subsequently sampled both edges. At Set Sizes 6 and 9, an exact *row-raster scan* required children to exhaust each row before entering the next while preserving the same horizontal direction across rows, whereas a *serpentine scan* required row-by-row coverage with alternating horizontal direction across successive rows. *Broader row grouping* required all locations within a row to be visited consecutively before transition to another row, irrespective of within-row ordering. *Local adjacency* was calculated as the proportion of consecutive first-visit transitions occurring between horizontally or vertically neighboring grid positions; diagonal transitions were not considered adjacent. *Revisiting* was defined as selecting any location or color identity that had already been sampled earlier in the same block and was therefore broader than the prespecified immediate-perseveration measure. Featural first-visit sequences were evaluated using the same spatial indices after color-to-code mapping, allowing identification of spatially indexed color sequences despite changing screen location. Sequences that did not exhibit canonical or row-grouped organization were characterized descriptively rather than treated as validated strategy categories. Within-child spatial-to-featural order similarity was quantified using Kendall rank association and compared with age-stratified permutation distributions generated by re-pairing the children across conditions. Paired spatial-featural differences were evaluated using Wilcoxon signed-rank tests, systematic-pattern carryover with Fisher’s exact tests, and age differences with Kruskal–Wallis or chi-square tests as appropriate. Because these analyses were not preregistered and were conducted after the primary analyses, they are considered exploratory and hypothesis-generating; *p* values are reported descriptively without multiplicity adjustment (see also Table S3 for descriptive coding).

### 2.5. Neural Data Analyses

The NIRS Brain AnalyzIR toolbox was used for fNIRS preprocessing and statistical analysis (Santosa et al., 2018). A block-design approach treated each task version (spatial or featural) at each set size as a distinct block. Prior to the downstream analyses, recordings underwent separate signal-quality control. Channel-level SNR pruning used a 20 dB threshold. Participants were retained for fNIRS analysis only if at least 80% of channels remained after SNR-based pruning, and scalp coupling index (SCI) exceeded 0.75 for all participants who reached this analytic stage. Each completed task block was additionally required to contain at least 30 s of usable fNIRS data after preprocessing and prior to individual and group level analyses. When combining jitter, selection response period, and instructions for each set size this was a reasonable criterion. Participant-, channel-, and block-level quality-control rules were applied in preprocessing before the downstream MATLAB/AnalyzIR analyses. The toolbox’s spatial PCA filter was then applied with ncomp = 0.80; this PCA parameter controls fractional spatial covariance removal and is distinct from the SNR, SCI, and 80% channel-retention criteria. Because spatial PCA can attenuate task-related variance when task responses covary with global physiology, this preprocessing choice is considered in the limitations and was not interpreted as uniquely optimal. Raw intensity was converted to optical density and then to hemoglobin concentration using the modified Beer–Lambert law with the executed AnalyzIR pathlength-factor setting PPF = 6. Recordings without usable stimulus events after cleaning were removed before subject-level estimation. Block duration varied across children, thus block-level estimates were bounded by the Start and Stop triggers. Subject-level effects were estimated with a general linear model (GLM). HbO and HbR were retained as complementary but distinct chromophore outcomes; they were not collapsed into a single statistical measure. The expected pattern of positive HbO with negative HbR was used only as a physiological consistency check when interpreting task-evoked responses. Statistical inference was conducted separately by chromophore/channel, with false-discovery-rate corrected *q* values used for multiple-comparison control.

**Figure 2:**
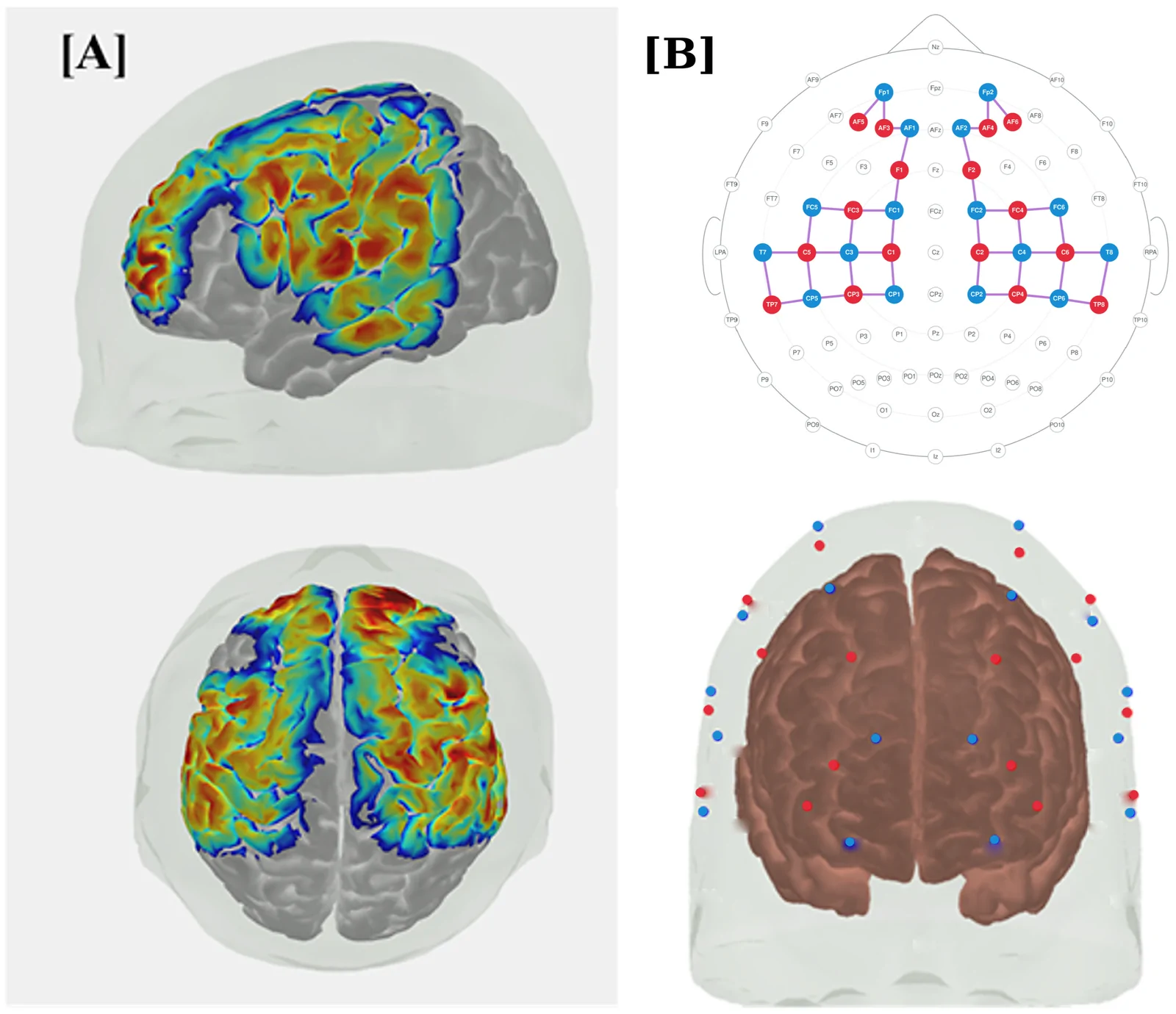
Sensitivity profile (**A**) and schematic of probe design (**B**). Channel-level cortical labels reflect the standardized cap alignment and probe sensitivity profile. Participant-specific digitization was completed for a subset of children (*n* = 31) and was less tolerated by the youngest children relative to the oldest. Anatomical labels should be interpreted as channel-to-region assignments via NIRStar EasyCap capping procedures.

Group-level fNIRS effects were evaluated with AnalyzIR mixed-effects models using first-level channel beta estimates and their covariance information (Santosa et al., 2018). Analyses were run separately by set size and chromophore. The final fixed-effects structure tested the condition (spatial vs. featural), age in years, symptom-defined ADHD risk group, condition × age, and condition × ADHD-risk interactions. Repeated observations from the same child were modeled with a subject-level random intercept; no random slopes were specified in the final model. In Wilkinson-Rogers notation, the final specification was beta ~ condition * age + condition * ADHD_risk + (1|subject). AnalyzIR uses first-level covariance information in weighted group-level estimation. The toolbox returns uncorrected *p* values and FDR-corrected *q* values for each chromophore/channel; *q* ≤ 0.05 was the threshold for reported neural findings. Set size was analyzed in separate models rather than entered as a repeated fixed factor because not all children advanced to Set Size 9. For probe design and coverage details see Figure 2. 

## 3. Results

Survey results are reported first, followed by behavioral efficiency (number of selections) and the prespecified perseveration measure. Neural results are then presented in a consistent order: spatial-condition effects, featural-condition effects, and symptom-risk-group effects, with group-level effects distinguished from age interactions. Behavioral and neural analyses were conducted separately for each set size. Neural results reported below reflect channels meeting the FDR-corrected threshold (*q* ≤ 0.05). Because spatial clustering and color-grouping sequences were not separately coded, the behavioral strategy results are limited to immediate perseveration. An exploratory post hoc sequence analysis then characterizes broader spatial and featural search organization, developmental differences in those patterns, and whether organization in a preceding spatial block carried over to the subsequent featural block.

### 3.1. Survey Results

Associations among child SDQ Hyperactivity scores, age, and parental ADHD-related symptoms were examined using Pearson correlations. SDQ Hyperactivity scores were not significantly related to age, *r*(198) = 0.104, *p* = 0.144. Paternal ASRS inattentive and hyperactive symptom scores showed small positive associations with child SDQ Hyperactivity, *r*(137) = 0.218, *p* = 0.010, and *r*(137) = 0.216, *p* = 0.011, respectively. Maternal ASRS scores showed positive associations with child SDQ Hyperactivity for both inattentive, *r*(197) = 0.381, *p* < 0.001, and hyperactive symptoms, *r*(197) = 0.363, *p* < 0.001. These associations provide convergent symptom information but do not establish a child or parent ADHD diagnosis.

### 3.2. Number of Selections Made

For each set size, repeated-measure analyses examined informative condition (spatial vs. featural/color), age, symptom-defined ADHD risk, and their relevant effects on the number of selections required to locate all treasures. For Set Size 3 (*N* = 200), children required fewer selections in the spatial condition (*M* = 3.47, *SD* = 1.16) than in the featural/color condition (*M* = 7.42, *SD* = 3.41), *F*(1, 195) = 91.19, *p* < 0.001, η_p_^2^ = 0.319. A significant main effect of age was also observed, *F*(1, 195) = 31.44, *p* < 0.001, η_p_^2^ = 0.139, with older children requiring fewer selections overall. The condition × age interaction was significant, *F*(1, 195) = 4.53, *p* = 0.035, η_p_^2^ = 0.023. Symptom-defined ADHD risk did not significantly predict selection efficiency at this set size.

For Set Size 6 (*N* = 197), children again required fewer selections in the spatial condition (*M* = 4.01, *SD* = 1.86) than in the featural/color condition (*M* = 10.41, *SD* = 4.02), *F*(1, 193) = 81.14, *p* < 0.001, η_p_^2^ = 0.296. A significant main effect of age was observed, *F*(1, 193) = 18.92, *p* < 0.001, η_p_^2^ = 0.089, with older children requiring fewer selections overall. The condition × age interaction was also significant, *F*(1, 193) = 7.71, *p* = 0.006, η_p_^2^ = 0.038. Symptom-defined ADHD risk did not significantly predict selection efficiency at Set Size 6.

At Set Size 9, 163 children began the condition and 162 completed both spatial and featural blocks. Within the complete-condition subset (*N* = 162), the main effect of informative condition was not significant, *F*(1, 157) = 0.57, *p* = 0.451, η_p_^2^ = 0.004, indicating no statistically reliable difference in selections between the spatial condition (*M* = 11.56, *SD* = 3.85) and the featural/color condition (*M* = 16.28, *SD* = 7.02) within the subset that advanced to this condition. A significant condition × age interaction was observed, *F*(1, 157) = 9.64, *p* = 0.002, η_p_^2^ = 0.058. Because Set Size 9 eligibility depended on successful completion of Set Size 6, these effects pertain to a selected higher-performing subset and should not be generalized to the full sample.

**Figure 3:**
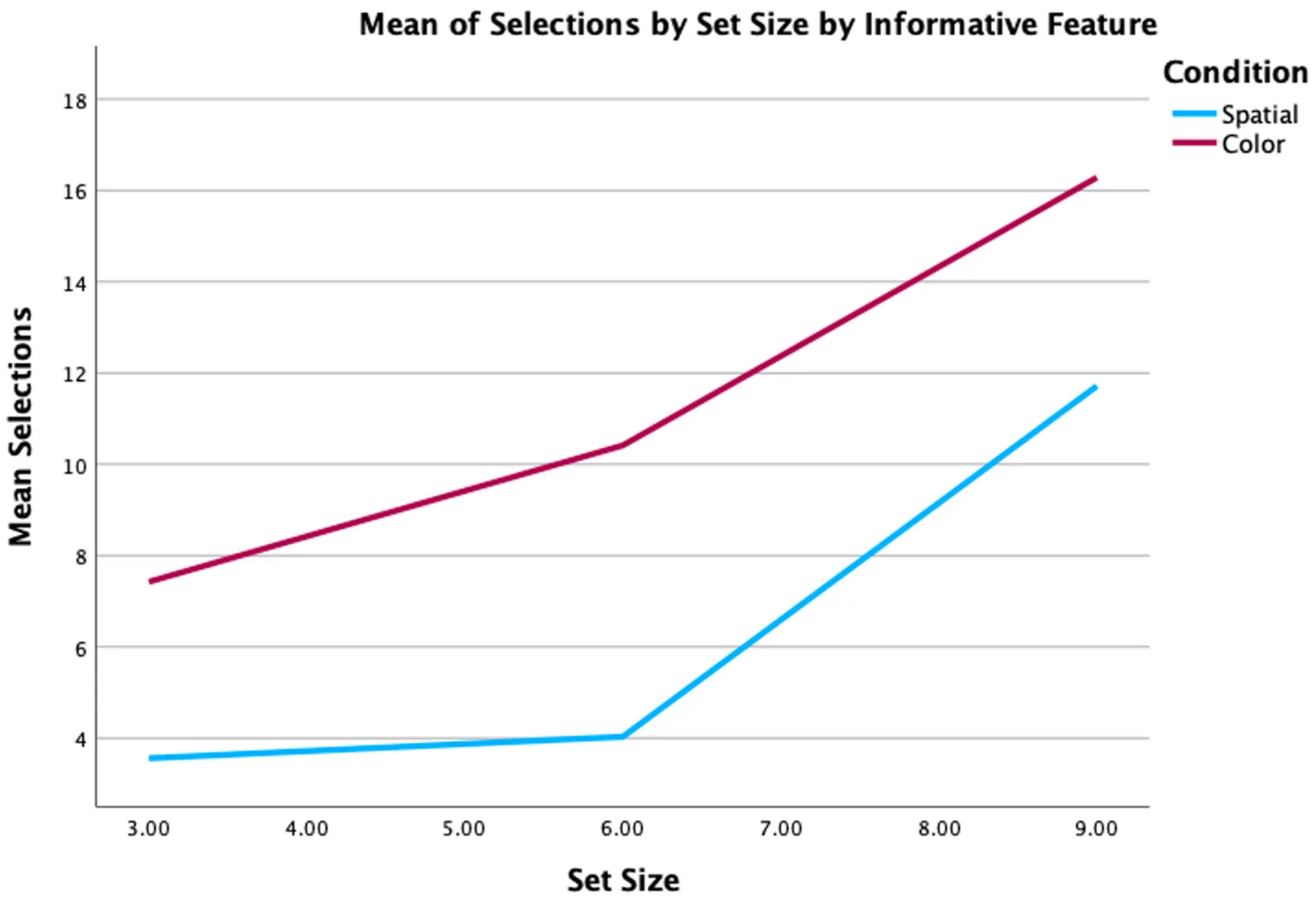
Mean number of selections required to locate all treasures across set sizes 3, 6, and 9 for spatial and color conditions. Each line represents one of the informative features.

Across Set Sizes 3 and 6, children required fewer selections when spatial location remained informative than when search depended on featural/color identity, and performance improved with age. At Set Size 9, the condition difference was no longer statistically reliable within the selected subset, although age-related effects remained. Across the reported set sizes, selection efficiency did not significantly differ by symptom-defined ADHD risk status (see Figure 3).

### 3.3. Perseveration Analysis

To examine developmental and symptom-risk differences in immediate repetition, we modeled perseverative selections using a generalized linear model with a Poisson distribution and log link. Age, symptom-defined ADHD risk status, and their interaction were entered as predictors. This analysis was intentionally restricted to immediate perseveration and does not represent a comprehensive analysis of all possible spatial or featural search strategies.

A significant main effect of age was found, Wald χ^2^(1) = 190.65, *p* < 0.001, indicating that younger children made more immediate perseverative selections than older children. There was no main effect of symptom-defined ADHD risk, Wald χ^2^(1) = 0.11, *p* = 0.740, and no age × risk interaction, Wald χ^2^(1) = 1.16, *p* = 0.281. Thus, immediate perseveration decreased with age regardless of risk status. These results support an age-related improvement in one component of search control, but they do not establish perseveration as the sole driver of performance change because other strategies (e.g., spatial clustering or color grouping) were not quantified.

#### Exploratory Post Hoc Search-Pattern Analyses

Given that the primary strategy analysis intentionally focused on immediate perseveration, we conducted an exploratory post hoc sequence analysis to determine whether children demonstrated broader organization in how they searched across the spatial and featural conditions. These analyses were not preregistered and should therefore be interpreted as descriptive and hypothesis-generating. Choice-level data from both cohorts were concatenated and harmonized to a common item-identity structure using the study coding schema. For spatial blocks, this allowed us to characterize patterns based on the physical organization of the display. For featural blocks, color identities were additionally mapped onto the corresponding coding-schema indices, allowing us to test whether children preserved a spatially organized search structure even when color, rather than location, was the informative feature.

At Set Size 3, children showed a highly recognizable spatial search pattern. Among 198 children with complete first-visit spatial sequences, 119 (60.1%) used a linear edge-to-edge sweep, moving left-to-center-to-right or right-to-center-to-left. An additional 62 children showed a center-first split, beginning at the center location before sampling the two outer locations, whereas 17 showed an edge-jump/nonlinear first-visit order. Featural search was more heterogeneous: among 198 complete featural first-visit sequences, 77 showed a serial color-code sweep, 58 began with the middle-coded color, and 63 showed a nonserial color order. Paired comparisons already showed greater spatial organization at this lowest load. Spatial sequences had greater canonical scan similarity (mean Kendall’s τ = 0.734) than featural sequences (mean τ = 0.593), Wilcoxon *W* = 1798.5, *p* < 0.001, and a greater proportion of locally adjacent transitions (spatial *M* = 0.801; featural *M* = 0.694), *W* = 1798.5, *p* < 0.001.

The distinction between spatial and featural organization became substantially clearer at Set Size 6. Among 186 complete spatial sequences, 86 (46.2%) were exact row-raster or serpentine-row scans, and 115 (61.8%) showed broader row grouping even when the within-row order did not conform perfectly to a canonical scan. Specifically, 57 children showed a row-raster scan, 29 showed a serpentine-row scan, and another 29 showed row-clustered search. By comparison, only seven of 181 complete featural sequences (3.9%) produced an exact canonical spatial scan when color identities were mapped back onto their coding-schema locations, and only 30 of 181 (16.6%) were broadly row grouped. Most complete featural sequences were instead classified as idiosyncratic color sampling (71/181, 39.2%) or color cycling/revisit-heavy search (61/181, 33.7%). Among the 173 children with complete sequences in both conditions, spatial sequences showed substantially greater canonical scan similarity than featural sequences (mean τ = 0.819 vs. 0.566), *W* = 1341.5, *p* < 0.001, and a greater proportion of locally adjacent transitions (*M* = 0.736 vs. 0.464), *W* = 783.5, *p* < 0.001.

The same dissociation was evident at Set Size 9. Among 141 complete spatial sequences, 75 (53.2%) were row grouped and 49 (34.8%) were exact row-raster or serpentine scans. In contrast, none of the 104 complete featural sequences constituted an exact canonical row/column scan and only two of 104 (1.9%) were row grouped. Of these complete featural sequences, 50 were characterized by idiosyncratic color sampling and 44 by revisit-heavy/cycling behavior; together, these patterns accounted for 90.4% of complete featural Set Size 9 sequences. For the 98 children with complete sequences in both conditions, the spatial search again showed greater canonical scan similarity than featural search (mean τ = 0.734 vs. 0.417), *W* = 380.5, *p* < 0.001. Local adjacency was likewise greater during spatial search (*M* = 0.702) than featural search (*M* = 0.392), *W* = 112.5, *p* < 0.001.

We next asked whether a child’s organization during the preceding spatial block predicted how that same child searched during the immediately subsequent featural block. The results did not indicate simple strategy carryover. Rank-order similarity between each child’s spatial first-visit sequence and subsequent featural first-visit sequence was essentially zero at Set Size 3 (mean Kendall’s τ = 0.003) and remained small at Set Sizes 6 and 9 (mean τ = 0.029 and 0.060, respectively). Age-stratified permutation analyses indicated that none of these observed within-child similarities exceeded the similarity expected when spatial and featural sequences were re-paired across the children: Set Size 3, two-sided *p* = 0.945; Set Size 6, *p* = 0.289; and Set Size 9, *p* = 0.246. Having an identifiable systematic spatial scan also did not increase the likelihood of subsequently showing a systematic spatially mapped featural scan at Set Size 3 (Fisher’s exact *p* = 0.552, *OR* = 0.82) or Set Size 6 (*p* = 0.720, *OR* = 0.73); at Set Size 9, no child with complete paired data produced an exact canonical featural scan. Treating featural organization continuously yielded the same conclusion. Children with versus without a systematic preceding spatial scan did not differ in subsequent featural scan similarity at Set Size 3 (mean τ = 0.580 vs. 0.612), Mann–Whitney *U* = 4475.0, *p* = 0.500; Set Size 6 (mean τ = 0.542 vs. 0.591), *U* = 3253.0, *p* = 0.132; or Set Size 9 (mean τ = 0.404 vs. 0.424), *U* = 1036.5, *p* = 0.702.

Age-related differences were minimal at Set Size 3. Canonical scan similarity did not differ across age groups in either the spatial condition, Kruskal–Wallis *H*(3) = 1.70, *p* = 0.636, or the featural condition, *H*(3) = 0.41, *p* = 0.937. A clearer developmental pattern emerged at Spatial Set Size 6. Canonical scan similarity differed by age, *H*(3) = 19.18, *p* < 0.001, as did local adjacency, *H*(3) = 22.76, *p* < 0.001. The percentage of complete sequences showing row-grouped organization increased from 37.9% among 2-year-olds to 65.9% among 3-year-olds and 75.4% among 4-year-olds before decreasing to 57.1% among 5-year-olds, χ^2^(3) = 12.32, *p* = 0.006, Cramer’s *V* = 0.26. Importantly, the age-5 reduction in rigid row grouping was not accompanied by increased inefficient revisiting. Mean spatial revisit rates declined from 0.305 at age 2 to 0.144 at age 3, 0.036 at age 4, and 0.055 at age 5, *H*(3) = 40.88, *p* < 0.001, suggesting that older children could search efficiently without consistently preserving a rigid raster-like sequence.

Spatial Set Size 9 produced an even clearer age gradient in row-based organization. Only 10.0% of complete sequences from 2-year-olds were row grouped, compared with 35.5% of 3-year-olds, 56.9% of 4-year-olds, and 69.4% of 5-year-olds, χ^2^(3) = 16.84, *p* < 0.001, Cramer’s *V* = 0.35. Although overall canonical scan similarity did not differ significantly by age, *H*(3) = 6.10, *p* = 0.107, local adjacency did, *H*(3) = 13.05, *p* = 0.005. Revisit rates again showed a strong developmental effect, declining from 0.486 among 2-year-olds to 0.207, 0.124, and 0.100 among 3-, 4-, and 5-year-olds, respectively, *H*(3) = 44.76, *p* < 0.001. Together, these findings suggest that increasing age was associated not simply with fewer repeated choices, but with increasingly organized coverage of the spatial array under higher working-memory demand.

The developmental pattern in featural search was notably different. At Set Size 6, neither canonical scan similarity, *H*(3) = 2.60, *p* = 0.457, nor local adjacency, *H*(3) = 0.16, *p* = 0.983, differed by age, and row-grouped featural search remained uncommon across age groups, χ^2^(3) = 0.75, *p* = 0.861. Featural revisiting did decline with age, from 0.450 among 2-year-olds to 0.384, 0.353, and 0.279 among 3-, 4-, and 5-year-olds, *H*(3) = 14.08, *p* = 0.003. At Set Size 9, even this age difference was no longer evident: featural revisit rates remained high at 0.447, 0.461, 0.404, and 0.405 across ages 2 through 5, respectively, *H*(3) = 2.67, *p* = 0.445. There were likewise no age effects on featural scan similarity, *H*(3) = 5.44, *p* = 0.143; local adjacency, *H*(3) = 1.89, *p* = 0.596; or row grouping, χ^2^(3) = 1.04, *p* = 0.792.

Several descriptive pattern classes emerged from these exploratory analyses that may be useful for future prospectively defined strategy work. Spatial search was characterized by a linear edge-to-edge sweep at Set Size 3; row-raster scanning, in which children exhausted successive rows in the same horizontal direction; serpentine row scanning, in which horizontal direction alternated across rows; row-clustered search, in which rows were exhausted as units without strict within-row ordering; and local-neighbor chains, in which children preferentially moved to nearby locations without forming a canonical raster pattern. A separate center-first split described Set Size 3 sequences beginning at the middle location before sampling both edges. Featural search was less readily characterized by spatial geometry. A small minority showed a spatially indexed color sequence, in which color identities reproduced a row-, column-, or neighbor-based spatial order when mapped back onto the coding schema. More commonly, the featural search reflected color cycling/revisit-heavy search or idiosyncratic color sampling. These labels are descriptive rather than validated strategy categories (see also Table S3).

### 3.4. Neural Results

**Figure 4:**
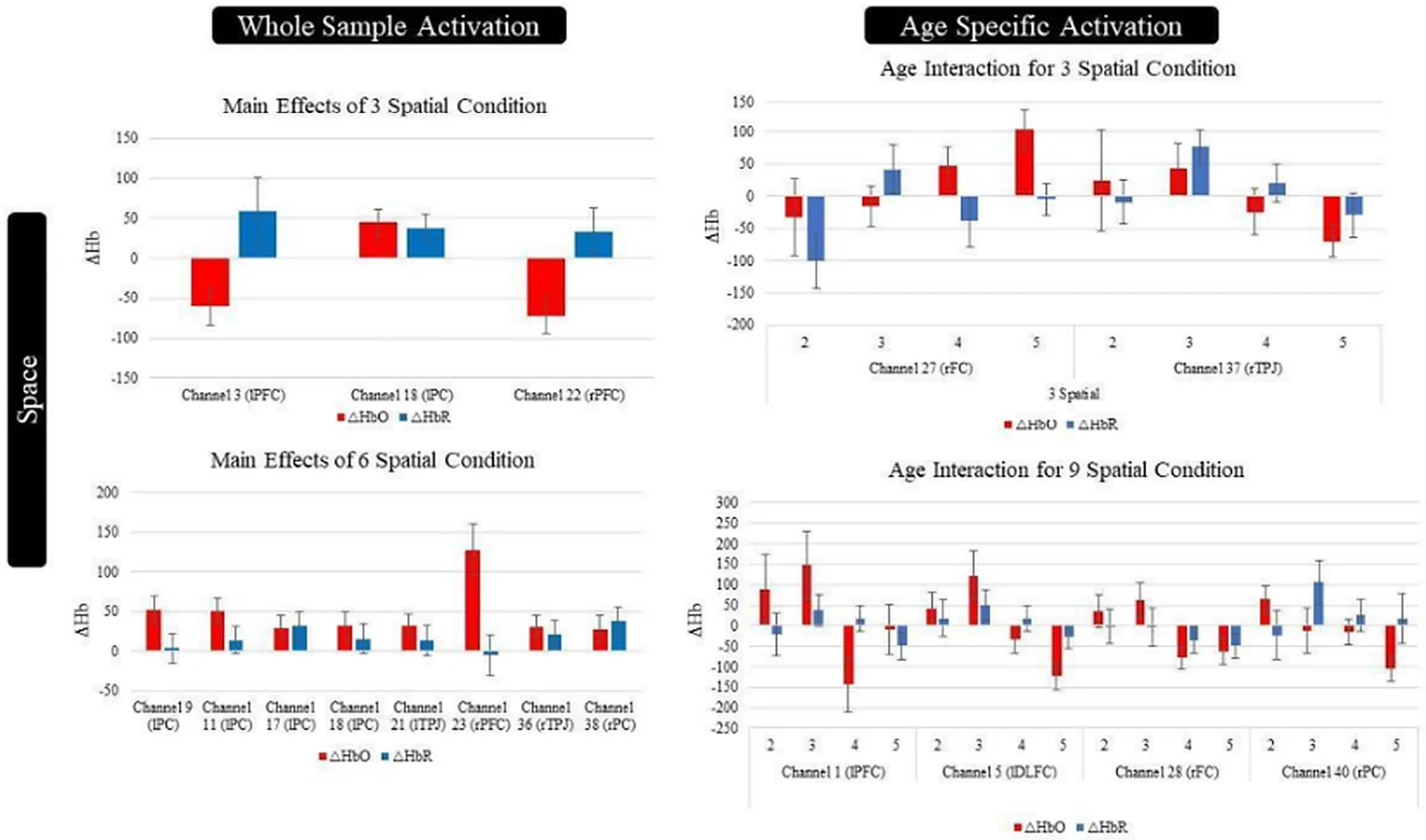
Significant spatial-condition channels and age-related effects by set size. Each displayed channel is labeled with its channel number and approximate cortical region based on the standardized sensitivity profile. Group-level effects and age interactions are shown separately; Set Size 9 effects should be interpreted cautiously because only an eligible subset completed that condition.

**Figure 5:**
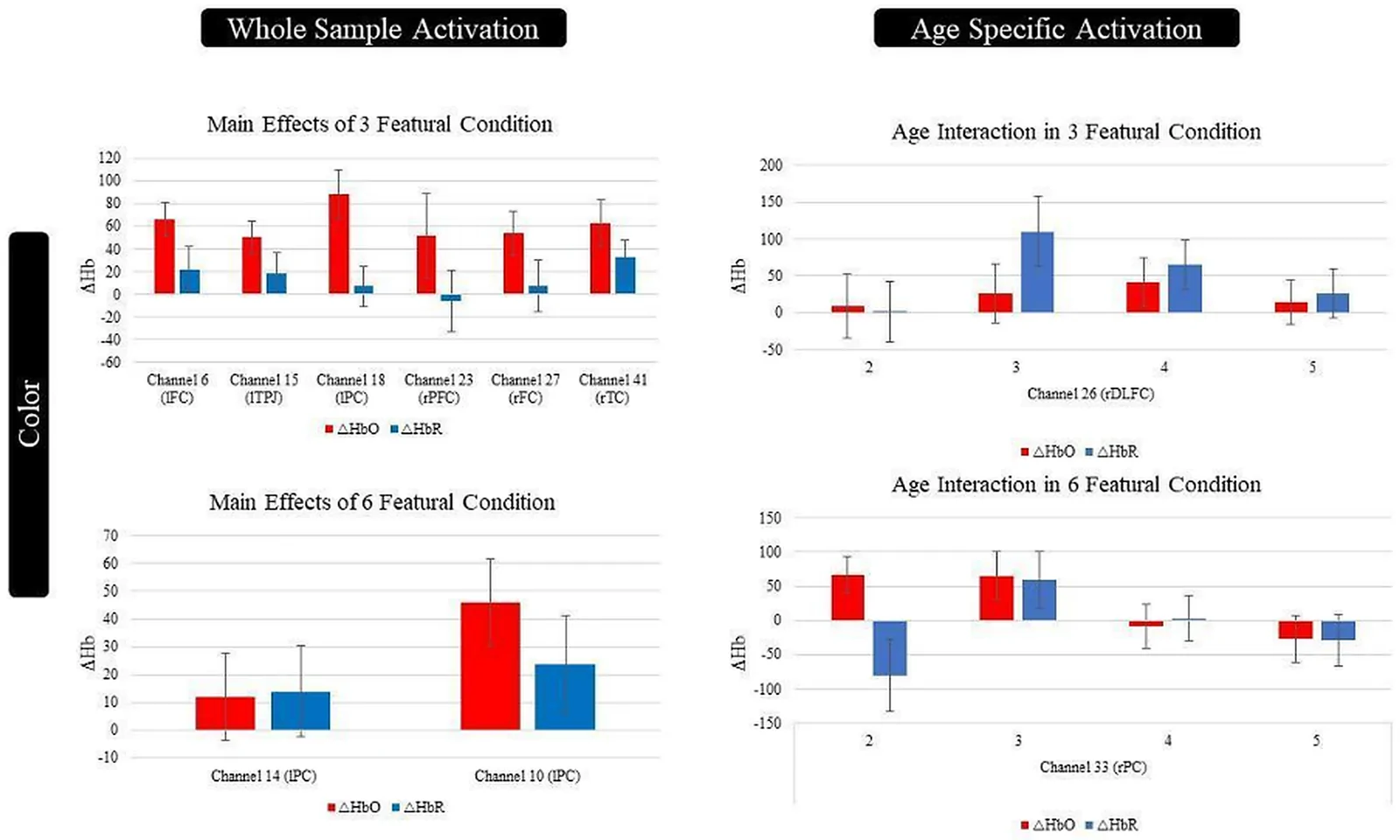
Significant featural/color-condition channels and age-related effects by set size. Displayed channels include channel number and approximate cortical-region label. Group-level effects are shown separately from age interactions; anatomical labels are based on the standardized probe sensitivity profile rather than participant-specific reconstruction.

**Figure 6:**
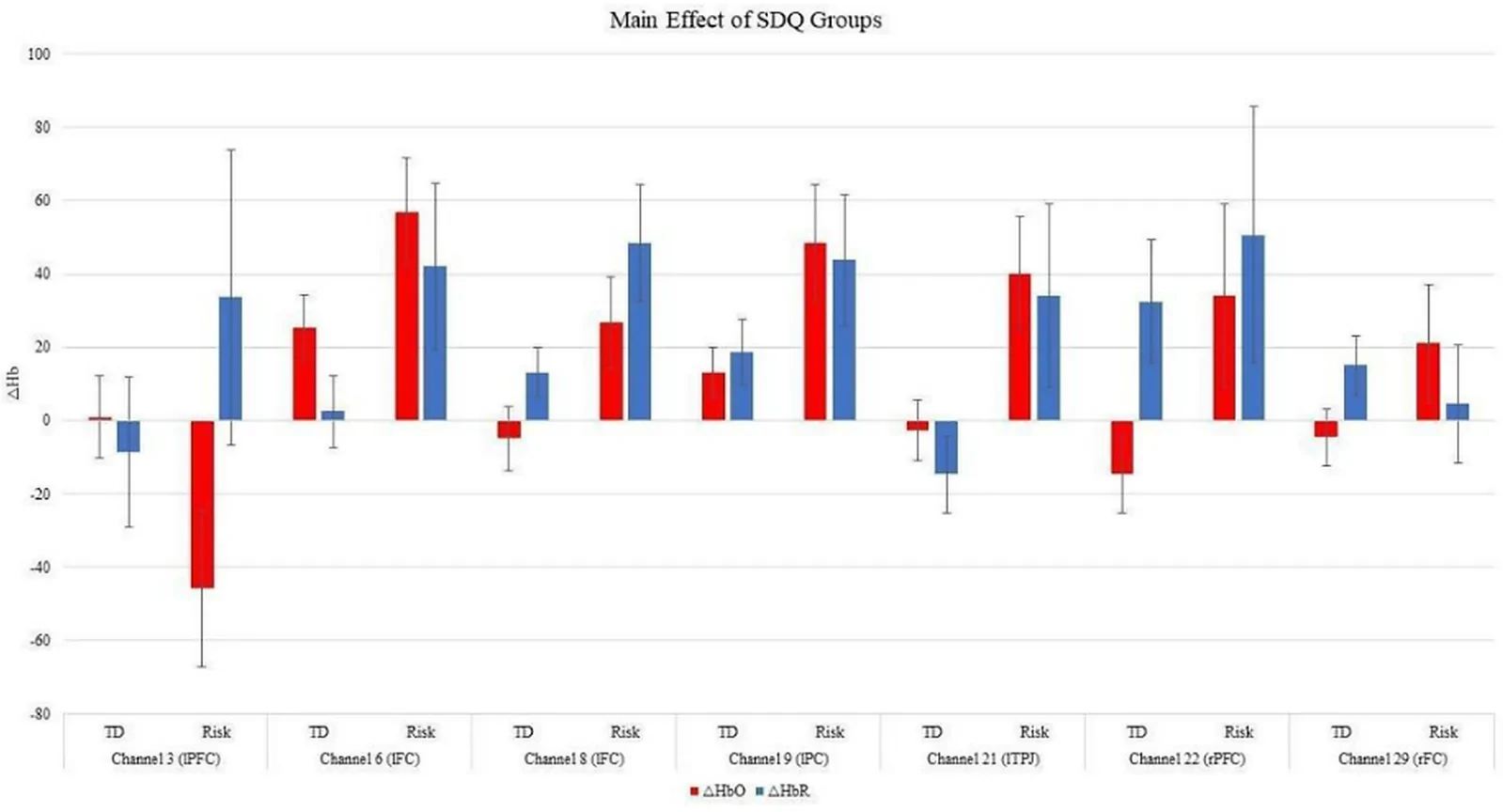
Frontotemporal–parietal recruitment differences between the elevated-symptom and lower-risk groups, controlling for age. The original plotting labels use “TD” for the lower-risk comparison group; here, this label should be understood as SDQ lower risk rather than a clinically verified diagnostic category.

Neural findings are organized below by spatial condition, featural condition, and symptom-defined ADHD risk. Within each subsection, group-level effects are described before age-related interactions to separate these inferential levels. Only channels meeting *q* ≤ 0.05 after multiple-comparison correction are discussed. Figure 4, Figure 5 and Figure 6 display the significant channel numbers with approximate regional labels. Abbreviations used in the figures include PFC (prefrontal cortex), FC (frontal cortex), DLPFC (dorsolateral prefrontal cortex), IFC (inferior frontal cortex), PC (parietal cortex), TPJ (temporoparietal junction), and TC (temporal cortex); l/r indicate left/right. Because individual digitization was not available for most participants, these labels describe the standardized probe sensitivity profile rather than participant-specific anatomy. A supplementary sex model produced one overlapping condition-level effect (right frontal channel 6 during Set Size 3 featural blocks), but sex was not retained in the primary models to maintain alignment with the behavioral analyses.

#### 3.4.1. Spatial Blocks

Spatial-condition results are summarized by the set size. At Set Size 3, the primary developmental effect was age related: younger children showed recruitment near the temporoparietal junction, whereas older children showed greater frontal recruitment. At Set Size 6, no age interaction survived correction; significant group-level responses were distributed across bilateral parietal/temporal channels and right prefrontal channels. At Set Size 9, recruitment was more variable across age groups, with younger children showing frontal and, for 2-year-olds, right parietal involvement, while older children did not show a consistent frontal pattern. Given the restricted Set Size 9 subset and broad variability, these high-load patterns are treated as descriptive and hypothesis-generating rather than evidence of a stable developmental cortical shift (Figure 4).

#### 3.4.2. Featural Blocks

Featural-condition results are summarized by the set size. At Set Size 3, significant group-level responses spanned left temporal, parietal, and prefrontal channels, while an age-related effect was observed in the right dorsolateral prefrontal cortex. At Set Size 6, group-level effects were concentrated in left parietal channels, with an age-related effect in right parietal cortex. No age interaction survived correction at Set Size 9. The overall pattern indicates distributed frontotemporal–parietal recruitment during featural search, but the extent and laterality of activation varied with age and set size; these results therefore should not be reduced to a single fixed network signature (Figure 5).

#### 3.4.3. Symptom-Defined ADHD Risk

Risk-group effects were evaluated after accounting for age. Table 1 summarizes channels showing significant associations between the SDQ-defined risk group and the task condition. The elevated-symptom risk group showed broader frontal recruitment than the lower-risk group in several blocks, including additional right frontal channels during featural conditions. Across featural blocks, significant responses included channels within the left frontotemporal–parietal montage, with additional right frontal involvement in the elevated-risk group. These are group-level associations in a symptom-defined sample; they do not establish an ADHD-specific neural marker, compensation, or neural inefficiency. The broader recruitment could reflect several nonexclusive mechanisms, including greater effort, less efficient processing, individual variability, or residual confounding. Because behavioral efficiency did not differ by risk group and no direct brain–behavior association was tested here, the functional meaning of the additional frontal recruitment remains unresolved.

## 4. Discussion

The current study leveraged a developmentally feasible, touchscreen-based Treasure Chest Task paired with functional near-infrared spectroscopy (fNIRS) to examine the early ontogeny of selective attention and working memory (WM), as well as risk-associated variation in cortical recruitment across early childhood. The findings provide evidence for developmental differences in spatial and featural processing, working-memory search, and neural recruitment that are compatible with dimensional-attention frameworks (Kerr-German & Buss, 2020). They also align with the core aim of the Behavioral Sciences Special Issue on advancing methods and theory in early development by offering a paradigm that is feasible for toddlers and preschool-aged children, sensitive to developmental differences, and informative about underlying neurocognitive processes (Kerr-German, 2025).

### 4.1. Selective Attention to Spatial vs. Featural Cues Across Development

Across set sizes within typical developmental capacity limits, children required fewer selections when spatial location was informative than when successful search depended on featural/color identity. This difference was evident at Set Sizes 3 and 6 and changed with age. At Set Size 9, the condition difference was no longer statistically reliable and variability increased. Advancement to Set Size 9 required successful completion of Set Size 6 and ≤18 selections on the shuffled featural Set Size 6 block; only 163 of the 197 children with complete Set Size 6 data began Set Size 9, and 162 completed both Set Size 9 conditions; this subset was selectively higher performing. Consequently, the apparent convergence of spatial and featural performance at the highest load cannot be distinguished cleanly from selection effects and reduced/generalizability of the restricted subset.

Developmental performance patterns were consistent with an age-linked shift in how children use spatial and featural information during the WM search. Younger children showed a clearer advantage when the spatial location remained informative, whereas featural/color search improved with age. Immediate perseverative selections also decreased with age, consistent with improving search control and updating. These changes are compatible with developmental accounts in which selective and flexible attention continue to mature across the preschool years (Buss & Kerr-German, 2019; Kerr-German & Buss, 2020), but the cross-sectional design does not establish within-child developmental trajectories or causal mechanisms.

### 4.2. Perseveration Decreases with Age but Represents One Search-Control Measure

Immediate perseveration declined strongly with age, regardless of symptom-defined ADHD risk. This provides a concrete index of age-related improvement in search control. Because the primary coding scheme captured only immediate repetition, the broader sequence analysis was conducted post hoc and is interpreted as exploratory rather than confirmatory. That analysis showed that developmental change extended beyond simply reducing repeated choices: spatial search became increasingly organized under higher loads, whereas featural search remained comparatively heterogeneous. Critically, the organization used in a preceding spatial block did not predict the child’s subsequent featural order, arguing against a simple strategy-carryover account. The Set Size 6 pattern in 5-year-olds further suggests that mature search efficiency need not require a rigid raster scan, because canonical row grouping decreased while revisiting remained very low. Overall, the exploratory sequence results are consistent with increasingly efficient spatial coverage emerging alongside age-related reductions in revisiting, while color-identity search remains more dependent on item-based updating when locations change. The proposed strategy labels are descriptive and require prospective validation.

### 4.3. Neural Recruitment Across Development

The neural data showed age- and condition-related differences in recruitment across the probe’s frontal, temporal, and parietal coverage. During lower-load spatial search, older children showed greater frontal recruitment while younger children showed more posterior recruitment, a pattern compatible with increasing top-down control. Featural search recruited a distributed frontotemporal–parietal set of channels, with age-related differences in right frontal/parietal regions depending on the set size. Recent longitudinal toddler fNIRS work similarly demonstrates developmental change in frontal–parietal recruitment during inhibitory control (Fiske et al., 2025), and child-friendly fNIRS measures of prefrontal connectivity relate to executive-function performance in early childhood (Eng et al., 2025). At the same time, our localization is channel-based rather than participant-specific for most children, so these results support broad regional interpretations rather than fine-grained anatomical claims.

The observed recruitment patterns are compatible with dimensional-attention accounts in which frontal systems increasingly bias processing toward task-relevant information with development (Buss & Kerr-German, 2019; Kerr-German & Buss, 2020). However, the current cross-sectional data do not demonstrate a literal transition from one neural mechanism to another within individual children. The findings instead identify age-associated differences in the cortical recruitment measured by this montage. This distinction is particularly important at Set Size 9, where selective advancement and greater variability limit group-level developmental inference.

### 4.4. Exploratory Symptom-Defined ADHD Risk and Neural Recruitment

Symptom-defined ADHD risk was not associated with significant differences in behavioral efficiency, but it was associated with differences in cortical recruitment. The elevated-symptom group showed broader frontal involvement in several featural blocks, including right frontal channels not observed in the lower-risk group. Several interpretations remain plausible. Additional recruitment could reflect compensatory control that supports comparable performance, but it could also reflect greater effort, less efficient processing, developmental heterogeneity, or residual physiological/measurement variance. Recent fNIRS studies of clinically diagnosed school-age children likewise report task-dependent prefrontal differences with heterogeneous direction and localization (Bian et al., 2025; Chu et al., 2025), underscoring the need for caution when assigning a single functional meaning to broader recruitment. Because the current study did not test a direct association between right-frontal response magnitude and perseverative errors, compensation remains a hypothesis rather than a demonstrated mechanism.

The absence of a behavioral risk-group difference alongside neural recruitment differences is therefore informative but not diagnostic. The present design is cross-sectional, the child’s risk classification is based on screening rather than clinical diagnosis, and environmental covariates were not included in the reported hemodynamic models. These data are best interpreted as evidence of risk-associated variation in neural allocation within this sample, not as an established early ADHD biomarker or as proof that neural differences precede later clinical impairment. Longitudinal follow-up with diagnostic outcomes and direct brain-behavior analyses will be required to evaluate that possibility.

### 4.5. Contributions to the “Toddler Data Desert” Literature

This study advances the Special Issue’s methodological goals by demonstrating a toddler-feasible touchscreen/fNIRS paradigm that preserves a common response structure while manipulating the information that supports working-memory search. The task spans lower and higher set sizes and can be used in children who are often excluded from paradigms requiring lengthy instructions or conventional button responses. Its principal contribution is therefore methodological and mechanistic: spatial and featural search can be compared within a common child-friendly framework while neural recruitment is measured concurrently. The Set Size 9 advancement rule and condition-specific timing differences, however, should be retained as design constraints when interpreting the paradigm.

At a theoretical level, the findings link dimensional selection, working-memory load, and age-related changes in search control without requiring a single global executive-function explanation. A broader lifespan perspective also places these early developmental findings within the larger problem of how executive function depends on changing neural and physiological constraints. This parallel should not be interpreted as a shared disease mechanism: early neurodevelopmental vulnerability and later-life neurodegeneration are biologically distinct. Rather, developmental fNIRS work on executive networks (Fiske et al., 2025; Eng et al., 2025), lifespan work on executive function in ADHD (Seidman, 2006), and later-life work on cellular homeostasis and neurodegenerative cognitive decline (Huang et al., 2026) jointly emphasize that cognitive performance reflects the integrity and organization of supporting systems at different points in the lifespan.

### 4.6. Limitations and Future Directions

Several limitations should guide the interpretation of the current results. First, Set Size 9 was administered only to children who completed both Set Size 6 conditions and did not exceed 18 selections on the shuffled featural Set Size 6 block. Of 197 children with complete Set Size 6 data, 163 began Set Size 9 and 162 completed both Set Size 9 conditions. This advancement rule enriched the Set Size 9 subset for stronger performers and creates selection bias. The diminished spatial-featural difference at Set Size 9 may therefore reflect true convergence under super-capacity demand, restricted sample composition, reduced power, or a combination of these factors; it should not be generalized to the full sample.

ADHD risk classification relied on a parent-report symptom cutoff rather than a child clinical diagnosis. The SDQ is supported for preschool use and recent work suggests promising psychometric properties even in 1- to 2-year-olds, particularly for externalizing symptoms, but validated clinical cutoffs remain less certain at the youngest ages (Croft et al., 2015; Byrne et al., 2024). However, only a subset of children in the second cohort were assessed with this measure for exploratory purposes only. Parental ASRS scores are also screening measures rather than diagnoses. Although documented familial ADHD diagnoses were used to enrich recruitment for some families, family diagnostic information was not uniformly available as the analytic grouping variable due to incomplete or unavailable familial medical records. Longitudinal work should determine whether the observed group differences predict later diagnosis or functional impairment in middle childhood.

Household income and parental education were collected but were not included as covariates in the reported hemodynamic models. The observed risk-group effects therefore cannot be interpreted as uniquely attributable to ADHD-related symptoms independent of socioeconomic or environmental variation. Available records permitted a descriptive summary of reported child-care/preschool settings (Supplementary Table S2), but they did not provide a standardized attendance/exposure variable or a definitive never-attended category for all children; preschool/nursery exposure was therefore not entered as an analytic covariate. Standardized analytic variables for nationality and child medication status were also not available in the current analysis materials.

Additionally, the preprocessing pipeline relied on spatial PCA with a fractional covariance parameter of 0.80. Although this setting follows the AnalyzIR PCAFilter convention, a formal sensitivity analysis against an alternative motion-correction pipeline (e.g., spline interpolation or TDDR) was not available in the manuscript materials. Future robustness analyses should determine whether the significant channels are preserved across preprocessing choices. Similarly, no direct correlation between right-frontal response magnitude and perseverative errors was tested in the current report, so compensatory interpretations remain provisional. The sequence-based strategy analyses were exploratory, post hoc, and not preregistered. The pattern taxonomy and associated age effects were derived from the observed choice sequences, and the reported *p* values were not adjusted for the number of exploratory tests. These findings therefore require replication using prospectively defined strategy categories and sequence metrics. Lastly, individual differences may be especially informative in this age range. The broad variability observed under high load and the dissociation between behavioral and neural group effects motivate future multimethod and person-centered analyses combining fNIRS with behavioral sequences, eye tracking, and/or EEG. Longitudinal designs are particularly important for distinguishing stable risk-associated patterns from transient developmental variability.

## 5. Conclusions

In summary, the Treasure Chest Task revealed age-related differences in selective attention and working-memory search across early childhood, including a spatial performance advantage at lower set sizes, improving featural search with age, and a robust decline in immediate perseveration. fNIRS results further showed age-associated differences in frontoparietal recruitment and symptom-risk group differences in frontal recruitment despite comparable behavioral efficiency. Because risk was defined by screening rather than diagnosis, localization was primarily channel based, and the design was cross-sectional, these neural differences should be interpreted as risk-associated group patterns rather than diagnostic biomarkers or demonstrated compensatory mechanisms.

The findings contribute to the Toddler Data Desert literature by demonstrating a feasible way to examine spatial versus featural information use, working-memory load, and cortical recruitment within a common touchscreen framework across ages 2–6 years. The results also illustrate why behavioral and neural measures can provide complementary information in early development while underscoring the need for longitudinal validation, precise anatomical registration, broader strategy coding, demographic sensitivity analyses, and clinically characterized follow-up samples before risk-associated neural patterns can be translated toward early identification or considered evidence of biomarkers.

## Figures and Tables

**Table 1:** Interaction between SDQ-defined symptom risk and condition for HbO during the treasure chest task.

Channel	*β*	*t*	*p*	Power
		3 Spatial: SDQ		
14	69.161	2.042	0.041	0.328
22	112.573	2.367	0.018	0.451
34	81.816	2.072	0.038	0.339
36	−66.561	−1.985	0.047	0.307
		3 Featural: SDQ		
26	84.172	2.172	0.030	0.376
		6 Spatial: SDQ		
3	−106.007	−2.227	0.026	0.397
27	−123.282	−3.478	0.001	0.839
		6 Featural: SDQ		
2	89.364	2.080	0.038	0.342
4	150.096	2.138	0.033	0.363
7	86.521	2.144	0.032	0.365
8	107.198	2.538	0.011	0.520
14	84.046	2.546	0.011	0.523
26	93.400	2.366	0.018	0.451
42	79.322	2.239	0.025	0.402
		9 Spatial: SDQ		
6	93.247	2.414	0.016	0.470
21	88.896	2.368	0.018	0.452
40	73.078	2.067	0.039	0.337
		9 Featural: SDQ		
18	−85.554	−2.232	0.026	0.399
20	121.792	2.188	0.029	0.382
32	85.085	2.166	0.031	0.373

## Data Availability

Due to the sensitive nature of the questions asked in this study, survey respondents were assured that raw data would remain confidential and would not be shared with identifying information. Deidentified neural and behavioral data will be shared upon request.

## Supplementary Materials

The following supporting information can be downloaded at: https://www.mdpi.com/article/10.3390/bs16101894/s1.
