# Supplementary material for: Probing Executive Function in the Toddler Data Desert: A Touchscreen fNIRS Study of Working Memory and ADHD-Related Symptoms
Source: Behav Sci (Basel). 2026 Oct 14;16(10):1894. doi: 10.3390/bs16101894 (PMC13701727; doi:10.3390/bs16101894)
Supplement: Supplementary file 1 [file behavsci-16-01894-s001.zip › behavsci-4543313-Supplementary.pdf]

Supplementary Descriptive Tables

Supplementary Table S1

Participant Retention Across Treasure Chest Task Stages by Age

Stage	Age 2	Age 3	Age 4	Age 5	Total *N*	Change	Retention
Enrolled in broader study	41	49	57	57	204	—	—
Completed both Set Size 3 conditions	38	48	57	57	200	-4	98.0%
Completed both Set Size 6 conditions	37	47	57	56	197	-3	98.5%
Started Set Size 9	24	37	51	51	163	-34	82.7%
Completed both Set Size 9 conditions	23	37	51	51	162	-1	99.4%

Note. Retention percentages are calculated relative to the immediately preceding stage. The reduction from Set Size 6 to Set Size 9 primarily reflects protocol-based non-advancement rather than conventional participant attrition. Separate fNIRS retention reflected child tolerance/continuation with cap wear and pre-analysis quality control. Channel-level pruning used an SNR threshold of 20 dB; participants entering the fNIRS analyses retained at least 80% of channels after pruning, and SCI exceeded .75 for all participants retained at this stage. Completed blocks also required at least 30 s of usable fNIRS data. Exact model-specific fNIRS Ns remain to be verified from the final analysis outputs.

Supplementary Table S2

Available Child-Care/Preschool Educational Settings by Age

Educational setting	Age 2	Age 3	Age 4	Age 5	Total *n*	% of *N*=200
Religious (Jewish, Lutheran, Catholic, and Other Christian)—Part-Day	0	1	7	6	14	7.00
Homeschool Families—Supplemental Pre-K	1	2	2	1	6	3.00
Montessori School	0	1	0	0	1	0.50
Private For-Profit School—Unspecified (e.g., Academies)	0	0	1	4	5	2.50
Private For-Profit Pre-K/Early Learning	1	0	3	0	4	2.00
Public Pre-K/Early Learning	1	4	3	3	11	5.50
University Research Early Learning Center	1	0	0	1	2	1.00

Note. Percentages use the Set Size 3 behavioral sample (N = 200). Categories reflect educational settings identifiable in the available records; they do not constitute a complete standardized attendance measure, and absence of a listed setting does not indicate that a child had no preschool/nursery exposure.

Supplementary Table S3

Operational Definitions for Exploratory Spatial and Featural Search-Pattern Analyses

Search measure or pattern	Operational definition	Scoring / classification
**First-visit order**	Ordered sequence in which each unique spatial location or color identity was selected for the first time within a block. Subsequent selections of an already visited identity were omitted from this sequence.	Sequence of unique first visits for each participant, condition, and set size.
**Linear edge-to-edge sweep**	At Set Size 3, the child began at one outer location, selected the center location next, and ended at the opposite outer location.	Exact sequence of Left → Center → Right or Right → Center → Left.
**Center-first split**	At Set Size 3, the child selected the center location first and subsequently sampled both outer locations.	Exact sequence of Center → Left → Right or Center → Right → Left.
**Edge-jump / nonlinear search**	At Set Size 3, the child began at an outer location but did not transition to the adjacent center location on the next first visit.	Examples include Left → Right → Center and Right → Left → Center.
**Row-raster scan**	At Set Sizes 6 or 9, the child exhausted all locations within one row before entering the next row and traversed successive rows in the same horizontal direction.	Exact canonical row-by-row sequence; completely reversed versions were treated equivalently.
**Serpentine-row scan**	At Set Sizes 6 or 9, the child exhausted each row before entering the next but alternated horizontal search direction across successive rows.	Exact alternating row-by-row sequence.
**Broader row grouping / row-clustered search**	All locations belonging to one row were visited consecutively before the child transitioned to another row, but the order of selections within a row was unrestricted.	Binary classification indicating whether rows were searched as contiguous units. Raster and serpentine scans constitute more restrictive forms of row grouping.
**Local adjacency**	Consecutive first visits occurred between horizontally or vertically neighboring grid positions. Diagonal transitions were not treated as adjacent.	Proportion of adjacent first-visit transitions: number of adjacent transitions divided by the total number of first-visit transitions.
**Local-neighbor chain**	A spatial sequence showed repeated preference for immediately adjacent locations without satisfying an exact raster or serpentine pattern.	Descriptive pattern label based on local-neighbor organization; not treated as

Search measure or pattern	Operational definition	Scoring / classification
		a separately validated categorical strategy.
**Revisit**	Selection of any location or color identity that had previously been selected at any earlier point within the same block.	Revisit proportion calculated relative to all valid selections within the block.
**Immediate perseveration**	Selection of the same spatial location or color identity on the immediately subsequent choice.	Prespecified primary search-control measure; distinct from the broader revisit metric.
**Spatially indexed color sequence**	During featural blocks, color identities were translated to their corresponding coding-schema spatial indices. A sequence was considered spatially indexed when the resulting first-visit order reproduced recognizable spatial organization despite physical rearrangement of the colored chests.	Descriptive classification based on mapped first-visit order, including row-, column-, or neighbor-based organization.
**Color cycling / revisit-heavy search**	Featural search was characterized by repeated returns to previously sampled color identities rather than progressive coverage of unvisited colors.	Descriptive characterization supported by the continuous revisit measure; no independent categorical cutoff was imposed.
**Idiosyncratic color sampling**	A complete featural first-visit sequence did not meet an exact canonical scan or broader row-grouping definition after color identities were mapped to coding-schema indices.	Descriptive residual category; not interpreted as a validated cognitive strategy.
**Spatial-to-featural order similarity**	Degree to which a participant's first-visit ordering in a spatial block corresponded to the ordering used in the immediately subsequent featural block.	Kendall's τ; values near 0 indicate little systematic correspondence, whereas increasingly positive values indicate greater preservation of relative ordering.
**Systematic-pattern carryover**	Whether use of a systematic spatial search pattern was associated with use of a corresponding systematically organized pattern in the subsequent featural block.	Binary association evaluated using Fisher's exact test.

Note. These measures were derived from exploratory post-hoc sequence analyses and were not preregistered. Spatial identities were defined by fixed screen position. For featural blocks, color identities were mapped to the corresponding study coding-schema indices to permit evaluation of spatial organization despite location rearrangement. Exact canonical patterns and continuous measures such as local adjacency, revisiting, and Kendall's τ were used for statistical analyses. Labels such as local-neighbor chain, color cycling/revisit-heavy search, and idiosyncratic color sampling are descriptive, hypothesis-generating characterizations rather than prospectively validated strategy categories. Immediate perseveration was the prespecified primary search-control outcome and differs from revisiting in that perseveration required repetition on the immediately subsequent selection.
